# Stain Deconvolution Using Statistical Analysis of Multi-Resolution Stain Colour Representation

**DOI:** 10.1371/journal.pone.0169875

**Published:** 2017-01-11

**Authors:** Najah Alsubaie, Nicholas Trahearn, Shan E. Ahmed Raza, David Snead, Nasir M. Rajpoot

**Affiliations:** 1 Department of Computer Science, University of Warwick, Coventry, United Kingdom; 2 Department of Computer Science, Princess Nourah University, Riyadh, Kingdom of Saudi Arabia; 3 Department of Histopathology, University Hospitals Coventry and Warwickshire, Coventry, United Kingdom; Universidade de Mogi das Cruzes, BRAZIL

## Abstract

Stain colour estimation is a prominent factor of the analysis pipeline in most of histology image processing algorithms. Providing a reliable and efficient stain colour deconvolution approach is fundamental for robust algorithm. In this paper, we propose a novel method for stain colour deconvolution of histology images. This approach statistically analyses the multi-resolutional representation of the image to separate the independent observations out of the correlated ones. We then estimate the stain mixing matrix using filtered uncorrelated data. We conducted an extensive set of experiments to compare the proposed method to the recent state of the art methods and demonstrate the robustness of this approach using three different datasets of scanned slides, prepared in different labs using different scanners.

## Introduction

Direct analysis of stain expressions is pivotal for many tasks in the field of digital pathology. Typically, chemical stains are applied to a tissue section in order to highlight particular areas of interest. In digital pathology, these markers continue to play a key role, and automated algorithms frequently use stain expressions in a similar way as part of their analysis. These algorithms normally require the estimation of each applied stain out of multi-stained tissue image. For instance, on a Haematoxylin and Eosin (H&E) slide the Haematoxylin staining has been used as a guide to detect the nuclei [1] and as a learning feature to perform deep learning for cell detection [2]. The estimation of each stain colour are also used by several stain normalisation algorithms in order to find the contribution of each individual stain to the final color variation before performing stain colour normalisation [3–5]. The problem of stain colour estimation out of multi-stained images is exacerbated by the fact that the colour of a stain may vary depending on factors such as the stain manufacturer, room temperature, and the exposure time, which are likely to vary between different histology labs [6]. Hence, stain separation (also called stain deconvolution) out of multi-stained images is an essential step in most of histology image analysis algorithms.

Stain deconvolution is the process of transforming a stained tissue section image from the normal RGB colour space into a series of stain channels. Each stain channel is a grayscale image, which represents the intensity of a particular stain expression across the original image. Stain deconvolution methods typically attempt to find an ideal stain matrix, a matrix that when multiplied to the RGB colour channels produces the desired stain channels. A stain matrix is composed of stain vectors, each vector representing the model colour of a particular stain from the original image. In some applications, a normalisation step is conducted to standardise the stain colour appearance in all of processed images before processing each stain colour [3, 7–9].

Ruifrok and Johnston’s colour deconvolution algorithm [10] was among the first in this field. The method outlined certain key principals that more recent methods continue to use, such as the use of stain matrices and the conversion of RGB colour channels into optical density space. The authors also provided sample stain matrices to separate certain popular stain pairs, such as H&E or H&DAB. It should be noted, however, that these stain matrices were optimised for a particular set of images under certain staining conditions, and generally stain matrices need to be tuned to the exact stain colours present in the histology images. As a result, we may not achieve adequate deconvolution if we apply present stain matrix to images with different staining conditions [3, 11, 12].

To address the need for image-specific stain matrices, a number of stain deconvolution methods have been developed to estimate a specific stain matrix for a given input image. These methods typically apply some statistical analysis to the colour channels of an image and reduce it into a series of stain vectors. An early method of automated stain matrix estimation was described by Macenko *et al.* [11] as part of a method of stain normalisation. Stain vectors were estimated by taking singular value decomposition (SVD) of the image data. Gavrilovic *et al.* [13] considered the problem in the Maxwellian chromaticity plane, assuming that by projecting pixels into this space, perceptually similar colours will appear close to each other. Ideally, pixels are expected to appear in groups corresponding to each stain with some division between them. Pixel groups are modelled as a Gaussian mixture whose parameters are determined using an Expectation Maximisation (EM) approach. Each stain vector is then estimated as the mean of its corresponding Gaussian distribution.

Rabinovic *et al.* [14] compared two stain deconvolution approaches, Non-Negative Matrix Factorisation (NNMF) and Independent Component Analysis (ICA). They showed that while NNMF performed better, neither method was sufficient to fully deconvolve the images. The study was performed on hyper-spectral images, rather than light microscopy images. Hyper-spectral imaging typically operates on a greater number of input channels, compared to the three-channel RGB images, which may limit the comparison that can be made between methods for the two modalities.

Other works have taken a supervised approach to stain deconvolution such as Khan *et al.* [3] and Alsubaie *et al.* [15]. These methods make use of a pre-trained stain classifier to identify the locations where each stain is present. Stain colours, and thus the stain vectors, are then estimated from these sets of classified pixels. However, for such approaches to be viable, good quality annotated training data must be available for a variety of stain types, which is often challenging to obtain.

Kather *et al.* [16] proposed using PCA to get the optimal representation of stain colours. This is achieved by projecting the first two PCA components on the plane created by the stain vectors estimated using the pre-estimated stain matrix [10]. However, PCA assumes orthogonality between the main components, which is not always the case, especially in correlated stain colours such as H and E. Also, as we described above, using pre-estimated stain matrix using [10] is not always the best choice to represent the variability in stain colours as it assumes a fixed stain vectors for H and E images.

Trahearn *et al.* [12] recently proposed a method of stain deconvolution using a variant of ICA. The method is based on the assumption that stain vectors can be modelled as independent components according to the ICA model. When ICA is applied, it is expected that pixels of the same stain will be distributed approximately along the principal axis of one of the independent components and pixels of different stains will be distributed along different principle axes. However, Trahearn *et al.* [12] show that in some cases the raw independent components do not provide adequate deconvolution. Thus, a correction step is applied in order to adjust the estimated independent components. The set of optimal stain vectors is found by minimising the mean of the distances between each pixel and its nearest vector, stopping when convergence is achieved.

In theory, ICA recovers the independent components in the mixture based on two assumptions: a) source signals are independent, and b) they have non-Gaussain distributions [17]. Independency among sources is a strong assumption that might not always be satisfied, and the success of ICA is significantly dependent on this assumption [18]. In this paper, we propose an algorithm for stain deconvolution of histology images using independent component analysis in the wavelet domain. In this approach, the condition of independency among sources is relaxed. Each colour channel of the input image is decomposed into a series of narrow sub-band images using decimated wavelet transform. Statistical analysis is performed for each sub-band to find the least Gaussian sub-bands. Finally, ICA is applied to the selected sub-bands to estimate the stain matrix. Performing stain deconvolution using only the least Gaussian sub-bands increases independence among the separated sources.

The proposed framework also utilises textural information in stain colour deconvolution. The coefficients of the wavelet decomposition embed textural features of the image content which when acquired from the three colour channels could be used to find the stain mixing parameters through the powerful statistical blind source separation algorithm ICA.

We demonstrate the effectiveness of the proposed method in three different experiments using H&E scanned slides from three datasets: colon cancer [2], breast cancer [19] and lung cancer. Colon and lung tissue slides were scanned in the University Hospitals Coventry and Warwickshire (UHCW) while breast tissue slides were scanned at the Pathology Department of the University Medical Center Utrecht, Utrecht, The Netherlands. Faced with these variations in the datasets including slide preparation and scanning procedures, the proposed method shows robust results compared to state of the art.

## Materials and Methods

### Ethics Statement

The colon and lung cancer tissue slides were anonymously collected from the University Hospitals Coventry and Warwickshire (UHCW) NHS Trust in Coventry, UK. The ethics approval for a larger digital pathology study associated with this one was obtained from the National Research Ethics Service North West (REC reference 15/NW/0843). We also have a written permission to use breast images derived from our previous contribution to the AMIDA2013 contest [19].

### Blind Source Separation Model

In the blind source separation model, each component of the signal mixture **x** = *x*_1_, *x*_2_, …*x*_*i*_, *i* > = 1 is represented as a linear combination of the source signals **s** = *s*_1_, *s*_2_, …*s*_*j*_, *j* = 1, 2, 3, …*r*, *r* > = 2 mixed by a mixing matrix **M** [22],

$$\left[\begin{array}{c} x_{1}\\ x_{2}\\ ..\\ x_{i} \end{array}\right]=\left[\begin{array}{c} m_{1,1}\;m_{1,2}\;...\;m_{1,j}\\ m_{2,1}\;m_{2,2}\;...\;m_{2,j}\\ ....\\ m_{i,1}\;m_{i,2}\;...\;m_{i,j} \end{array}\right]\left[\begin{array}{c} s_{1}\\ s_{2}\\ ..\\ s_{j} \end{array}\right]$$ (1)

or simply,

x=Ms (2)

The objective is to separate the mixture such that the original source signals are recovered. One way of doing this is to find the mixing matrix **M** using Independent Component Analysis (ICA). ICA can recover source signals only if they are statistically independent. It separates the mixture by transforming it into a linear combination of components that are as non-Gaussian as possible. However, the assumption of independency among sources is precarious [20].

In the problem of stain colour deconvolution, sources are the original stains and the observed signals are the stain mixture. Therefore, we can show the dependency between different stain colours by comparing stain vector of each stain using two different staining contexts: In the first context, only one stain colour is applied, in the second context two stain colours are applied to the tissue as shown in [21] and [13]. It has been shown that the estimated stain vector for slide with only one stain colour, i.e, either H or E is different from the corresponding stain vector estimated from slides with multiple stains, for example H&E. Therefore, stain colours are actually not independent of each other and presence of one stain affect the others.

### Sub-band Independent Component Analysis

In order to reduce independency between the source signals, a linear filtering operator is applied such that the independent subcomponents are allowed to pass through. To explain that, consider **s**_*F*_ as the observation signals after applying a filtering operator *F*, i.e.

$$s_{F}=F(s)$$ (3)

Since **s** is linear combination of independent components and **M** is constant matrix. Then, we can similarly express **x**_*F*_ as follows,

$$x_{F}=F\left(s\right)=F\left(\text{Ms}\right)=MF\left(s\right)=Ms_{F}$$ (4)

**Fig 1:**
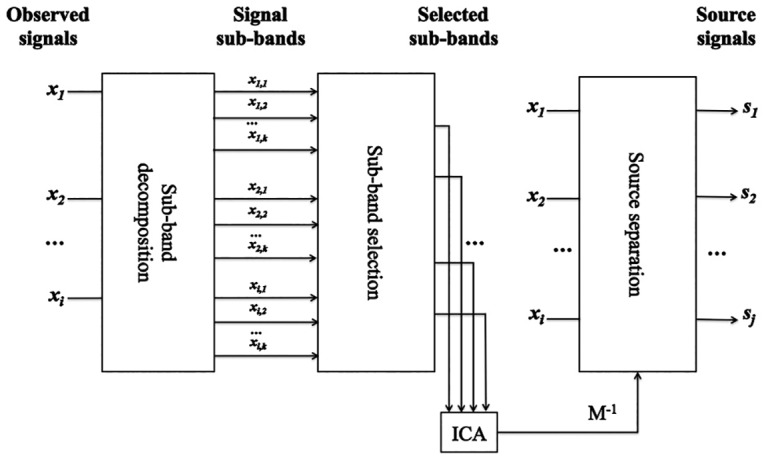
Blind source separation using sub-band decomposition with ICA. A filtering operator is applied to the observed signal *x*_*i*_ to extract independent and dependent sub-bands. The unmixing matrix is then estimated using a selected set of sub-bands which maximises independency among sources.

Thus, by applying ICA to the filtered observations **x**_*F*_, we can find the mixing matrix **M** which is the same matrix used to mix the raw signals. Therefore, we can apply **M** to the original data in Eq (2) to separate the mixture and estimate source signals, see Fig 1 for an illustration. More details about sub-band ICA can be found in [18, 22–24].

In the proposed method, observed signals are decomposed into several subbands using wavelet filters [25, 26]. The objective is to find which of the narrow sub-band signals provide independency among the sources, which can be achieved by selecting the sub-bands with the least Gaussian distribution [24]. This originates from the central limit theorem which states that the sum of independent signals tends to have a Gaussian distribution more than the original signals. Non-Gaussianity can be measured statistically using kurtosis, *l*_1_ norm, or *l*_2_ norm [27]. In our experiments, we find that kurtosis gives the most reliable measure of Gaussianity. In the following section, we describe the proposed framework which includes the filtering method and sub-bands selection criteria.

### Stain Matrix Estimation

**Fig 2:**
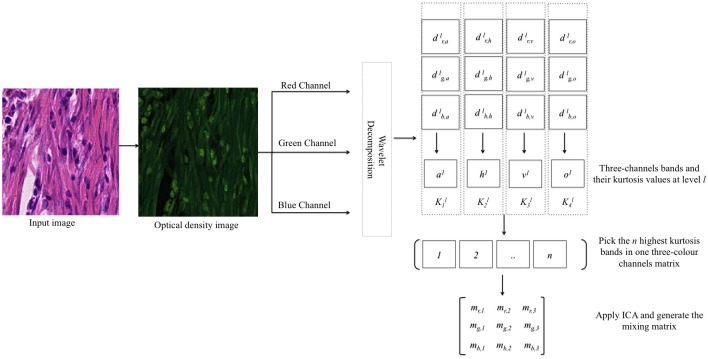
Block diagram of the proposed method.

We propose the use of multi-resolution representation of the input image, which is generated by decimated wavelet decomposition [25, 26] to find the mixing matrix. A block diagram of the proposed method is shown in Fig 2. According to the Beer-Lambert law, there is an exponential relationship between the amount of absorbed light **N** and the intensity of the transmitted light **I**, as given by the following equation,

$$I=I_{0}e^{-MN}$$ (5)

where *I*_0_ is the intensity of the incident light. In histology images, **M** is the concentration of stain colour represented as a vector of RGB components. In this paper, we refer to **M** as the mixing matrix. **N** is the amount of the absorbed stain at each pixel of the image which we refer to as density map of each applied stain.

The input image **I** is converted to the Optical Density space (OD) as follows,

$$D=-\log\left(\frac{I}{I_{0}}\right)$$ (6)

where **D** is the histology image in the OD space. Combining Eqs (5) and (6), we can express **D** as follows,

D=MN (7)

The task is to find the stain density maps **N** = *n*_1_, …*n*_*i*_, where *i* = 1, 2, 3 corresponds to the intensity of H stain, E stain, and the background for each pixel. Even though we assume that there are two stain colours in the image, the algorithm is applicable to images with more than two stains.

Given the optical density image as a three-rows matrix **D** = [*d*_*r*_ *d*_*g*_ *d*_*b*_]^*T*^, where each row corresponds to one colour channel, we decompose each colour channel into its sub-bands $d_{a}^{l},d_{h}^{l},d_{v}^{l},d_{o}^{l}$, where *l* = 1, 2, …*L*, *L* is the level of decomposition in the wavelet transform and *a*, *h*, *v*, and *o* denote approximation, horizontal, vertical and diagonal sub-bands of *d* at each level of decomposition.

For each level *l*, four three-dimensional sub-bands *a*^*l*^, *h*^*l*^, *v*^*l*^, *o*^*l*^ are composed as follows:

$$\begin{array}{c} a^{l}=\left[d_{r,a}^{l}\;d_{g,a}^{l}\;d_{b,a}^{l}\right]\;\;\;h^{l}=\left[d_{r,h}^{l}\;d_{g,h}^{l}\;d_{b,h}^{l}\right]\\ v^{l}=\left[d_{r,v}^{l}\;d_{g,v}^{l}\;d_{b,v}^{l}\right]\;\;\;o^{l}=\left[d_{r,o}^{l}\;d_{g,o}^{l}\;d_{b,o}^{l}\right] \end{array}$$ (8)

A normalisation step is applied to each sub-bands so that it has zero mean and unit variance. This is required to ensure that all the values are in the same scale before performing the non-Gaussianity comparison. We use kurtosis *K* to measure Gaussianity for each sub-band in Eq (8).

For a Gaussian distribution, *K* is equal to zero. Thus, we select sub-bands that maximise |*K*|. Each one of the selected sub-bands is reshaped into a three-rows matrix, where each row is one colour channel. Finally, selected sub-bands are concatenated to each other horizontally to form a single matrix **D**′ of size 3 × *p* where *p* is the total number of pixels in all the selected sub-bands. We selected 20 sub-bands from all five levels of decomposition and ordered them based on their kurtosis values. Then, we apply ICA to **D**′ to find the mixing matrix **M** as follows,

$$M=\left[\begin{array}{c} \;m_{r,1}\;\;m_{r,2}\;\;m_{r,3}\;\;\\ \;m_{g,1}\;\;m_{g,2}\;\;m_{g,3}\;\;\\ \;m_{b,1}\;\;m_{b,2}\;\;m_{b,3}\;\; \end{array}\right]$$ (9)

where *m*_*r*,*i*_, *m*_*g*,*i*_, and *m*_*b*,*i*_ are the mixing parameters of the red, green and blue colour channels for *H*, *E*, and background for *i* = 1, 2, 3, respectively.

Finally, stain colour distribution in the OD space is generated using the inverse of Eq (7). Therefore, we multiply the inverse of **M** with the original OD image **D**.

$$N=M^{-1}D$$ (10)

In contrast to most of the existing algorithms which rely only on colour information to perform deconvolution, the proposed approach automatically estimates the mixing matrix in Eq (2) by incorporating colour and texture of histology images. Using a filtered image rather than the OD image to find the stain matrix has several advantages: First, it only uses filtered and independent observations to reduce the contamination of signals when estimating stain matrix. Second, it takes texture information into account which is correlated with stain colours in histology images.

## Results

### Datasets

**Fig 3:**
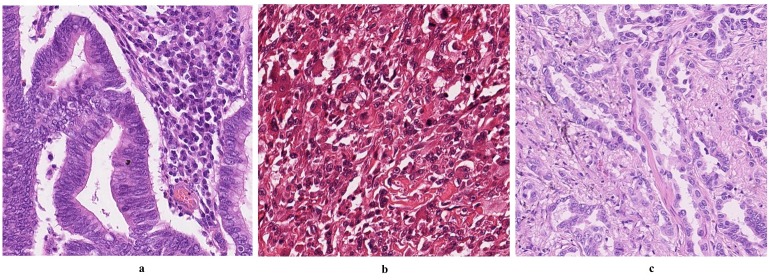
Sample images from the datasets used in our experiments. Images a, b, and c are samples from colon, breast, and lung cancer images, respectively. All of the datasets are H&E stain images. One can notice the huge variation in the colour appearance as they are applied to different tissue types and processed in different labs using two different scanners. These variations in colour appearance are really challenging for most of the stain deconvolution algorithms.

Stain chemicals bind differently to different tissue types. Therefore, it is essential to evaluate a stain deconvolution algorithm using different tissue types. We have also considered the variation in stain colour consistency by collecting images that have been scanned in two different labs. For the first and third datasets, automated H&E staining machine used was Tissue-Tek Prisma by Sakura joined to the coverslipping machine, Sakura Finetek Europe B.V. KvK / Chamber of Commerce Leiden 28065449. A sample image from each dataset is shown in Fig 3. In each dataset, we have selected a number of visual fields.

1. Colon Cancer Histology Images:
  The first dataset consists of seven colon cancer whole-slide images from different patients stained with H&E and scanned at 20× magnification by Omnyx VL120 scanners at UHCW. For each whole-slide image, two non-overlapping images of size 500 × 500 pixels are extracted from the same visual field. Selected visual fields represent areas with different stain colour distributions. They also include regions containing both tumour and non-tumour tissue.
2. Breast Cancer Histology Images:
  The second dataset comprises of three breast cancer whole-slide images from different patients stained with H&E. The slides have been scanned at 40× magnification by Aperio ScanScope XT scanners at the Pathology Department of the University Medical Center Utrecht, Utrecht, The Netherlands. The dataset has been published as part of the MICCAI contest on the Assessment of Mitosis Detection Algorithms (AMIDA2013) [19]. For each whole-slide image, we selected two non-overlapping images of size 2,000 × 2,000 pixels.
3. Lung Cancer Histology Images:
  The third dataset consists of two lung cancer whole-slide images from two different patients. Slides are stained with H&E and scanned at 40× magnification by Omnyx VL120 scanner at UHCW. For each whole-slide image, two non-overlapping images of size 2,000 × 2,000 pixels are extracted from the same visual field.

### Evaluation

In this section, we provide both quantitative and qualitative assessments of the proposed algorithm, in comparison to some of the recent existing methods of stain deconvolution. For the quantitative analysis, we perform three different experiments: First, we evaluate the accuracy of the estimated stain vector representing the concentration of the applied stain. Next, we evaluate the accuracy of the estimated density map, which is related to the amount of absorbed stain colour at each pixel. Third, we assess the performance of a nuclei detection algorithm [2, 28], which uses the H channel as a learning feature. In the following sub-sections, we describe each experiment and show the associated results. All data used in this experiments and the code files are placed in the supporting document file S1 File attached with this manuscript.

#### Evaluating the Estimated Stain Matrix

The stain matrix defines the principal colour of each applied stain. Each vector of the stain matrix represents the RGB values within the OD space for one stain colour. It is essential to find an accurate stain matrix, as it will affect the estimated stain colour intensity. However, we need a reference ground truth stain matrix in order to evaluate the quality of an estimated stain matrix. Thus, for each visual field, we have generated the ground truth stain matrix as follows: A set of pixels are selected from all images in the visual field. Note that pixels are selected based on their biological structure rather than their stain colour. This means that, for Haematoxlyin, we only selected pixels that belong to nuclei. For Eosin, we selected pixels that belong to cytoplasm. For a given stain, we calculated its stain vector by taking the median of each colour channel across the selected pixels in OD space. The final stain matrix is generated by horizontal concatenation of all the stain vectors. We then compute the Euclidean distance between the estimated stain vector and the ground truth stain vector.

**Fig 4:**
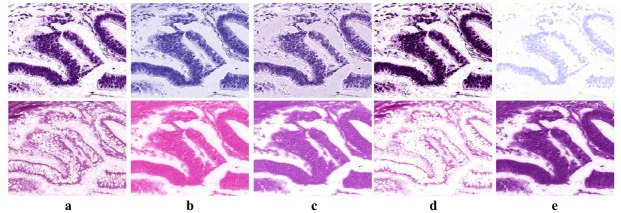
Stain colour deconvolution results for a colon tissue image. The first and second rows show the H and E channels, respectively for each algorithm. Column a, b, c, d, and e shows the deconvolution results for the Proposed method, Ruifrok and Johnston [10], Macenko *et al.* [11], BCD [13],and CA [12], respectively. There are two factors one could look at when evaluating the qualitative separation results, first: the accuracy of the separation and second:the stain colour estimation. In this sample image, we can see that the proposed method is achieving good stain separation and stain colour estimation compared to the other methods.

Results shown in Table 1 reveal that the proposed method outperforms Macenko *et al.* algorithm [11] for H channel estimation by around 2–7% for colon and breast datasets. For the E channel estimation, we report an improvement by 11–92% for colon and lung datasets, respectively. Ruifrok and Johnston algorithm [10] produce a higher euclidean distance of the H and E estimation by 4–17% and 11–27% compared to the proposed method for colon, breast and lung datasets respectively. For the BCD [13], we noticed the tendency of dark intensity colour for all of the datasets. The estimation error for BCD is 25–35% for H and 24–30% for E larger than the error reported for the proposed method in all the three datasets. This is because the algorithm is mainly concerned about the deconvolution more than the stain colour estimation. This is clear in the separation results achieved by [13], see Fig 4.

**Table 1:** Euclidean Distance between the estimated stain matrix and the ground truth. The median of the Euclidean distances for each method is shown in the last two columns. Last row shows the median of the Euclidean distances for all methods to highlight the significance of the best achieved median values.

	Colon Dataset		Breast Dataset		Lung Dataset		Median	
	H	E	H	E	H	E	H	E
**Proposed**	**0.0623**	**0.0871**	**0.0537**	0.0585	0.1377	**0.0779**	**0.0592**	**0.085**
**Macenko** ***et al.*** [11]	0.0774	0.2002	0.1202	**0.0488**	**0.0936**	0.9949	0.0971	0.4146
**Ruifrok and Johnston** [10]	0.1980	0.2017	0.2201	0.3150	0.1826	0.1917	0.2002	0.2361
**BCD** [13]	*0.3789*	0.3282	0.4027	0.3606	0.3897	0.3655	0.3905	0.3514
**ICA** [12]	0.2795	*0.3349*	*0.5219*	*0.5607*	*0.4081*	*1.2451*	*0.4032*	*0.7136*
**Median**	0.198	0.2017	0.2201	0.315	0.1826	0.3655	—	—

In order to find the effect of number of sub-bands in our proposed method, we run an experiment on the datasets described above. We performed this experiment using 5, 10, 15, and 20 number of sub-bands. Results in Table 2 show the Euclidean distance for the estimated H and E stain vectors and the ground truth. We found that for most of the datasets, using all the 20 sub-bands improves the accuracy of the generated stain vectors. For the first dataset, using 5 and 20 sub-bands generated closest stain vectors to the ground truth with a distance between them of 0.02. For the third dataset, a number of 10 and 20 sub-bands gives the highest accuracy with Euclidean distance of 0.06. Similarly, 20 sub-bands gives the highest accuracy for the second dataset.

**Table 2:** Euclidean Distance between the estimated stain matrix and the ground truth. Stain matrix is estimated using the proposed method by changing the number of selected sub-bands.

	Number of sub-bands							
	5		10		15		20	
	H	E	H	E	H	E	H	E
**Colon Dataset**	**0.0467**	0.0938	0.1113	0.0877	0.1316	0.1051	0.0623	**0.0871**
**Breast Dataset**	0.1490	0.1519	0.1077	0.1458	0.1304	0.1415	**0.0537**	**0.0585**
**Lung Dataset**	0.0589	0.0980	0.0457	0.0850	**0.0372**	0.0925	0.1377	**0.0779**

#### Evaluating Density Map Estimation

**Fig 5:**
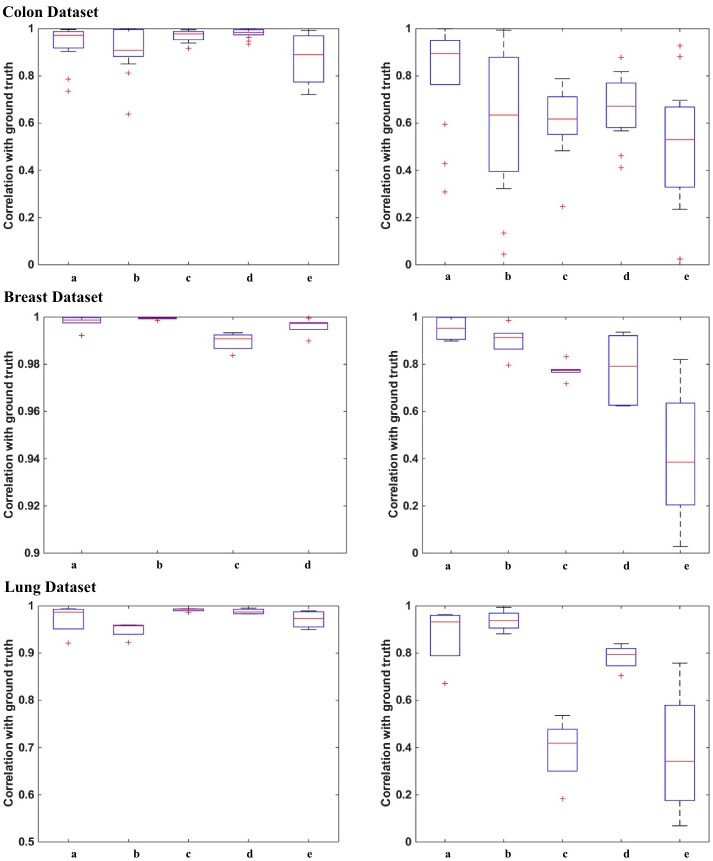
Correlation between the density maps and the ground truth. Indices a, b, c, d, and e of the x-axis show the correlation results for the Proposed method, Macenko *et al.* [11], Ruifrok and Johnston [10], BCD [13],and ICA [12], respectively. Due to the high difference in the correlation margin between ICA and the other algorithms in the H density estimation for the second dataset, ICA has been removed in order to make the correlations of the other algorithms noticeable.

**Fig 6:**
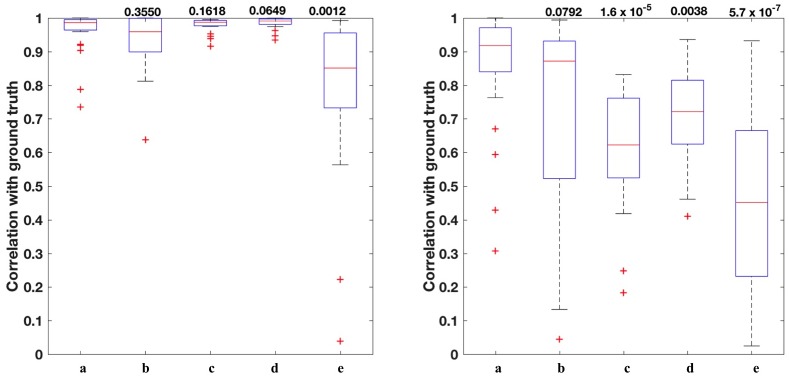
Correlation between the density maps and the ground truth with the associated *p-values* above each method for the H (left) and E (right) stains in all the three datasets. Indices a, b, c, d, and e of the x-axis show the correlation results of among all datasets for the Proposed method, Macenko *et al.* [11], Ruifrok and Johnston [10], BCD [13],and ICA [12], respectively Notice that most of the proposed methods perform similarly in estimating H satin (left). However, the weaker stain (E) is more challenging to estimate (right). Proposed method keeps its performance in estimating Eosin stain with mean significance of *p-value* < 0.05.

**Fig 7:**
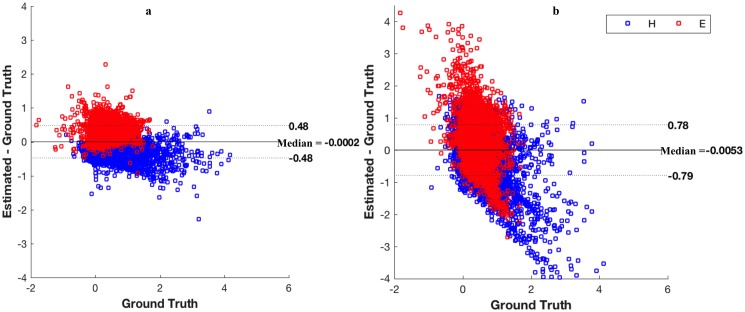
Bland Altman plot for the proposed method (left) and ICA [12] (right) for H and E stains using all datasets. Same randomly selected pixels are plotted from all three datasets by running the proposed method and ICA [12]. Median of agreement is -0.002 for the proposed method and -0.005 for [12]. Limits of agreements for the proposed method is [-0.48, 48] compared to [-0.79, 0.78] for ICA [12].

A density map shows the distribution of a particular stain across the section. For each image, the ground truth density map is generated using the corresponding ground truth stain matrix. The correlation coefficients between the estimated and ground truth density maps for each of the three datasets are shown in Fig 5. Figures show that the weaker stain (Eosin) becomes more challenging to estimate for most of the algorithms. Although most of the algorithms are performing comparatively when it comes to estimating the stronger stain, Haematoxylin in this case, only the proposed algorithm and Macenko *et al.* [11] are able to provide the most satisfactory results for Eosin with a median correlation of 89% and 63% respectively for colon tissues 95% and 91%, respectively for breast tissue, and 93% and 94%, respectively for the lung tissues. Correlation values for all datasets with the corresponding *p-values* are shown in Fig 6. To particularly investigate the improvement of the estimated stains when applying ICA to the filtered image rather than the raw OD image, Fig 7 shows a Bland Altman plot for the same randomly selected pixels for both proposed method and ICA [12] in all the three datasets. It is noticeable that in the proposed method most of the pixels are lying within the limits of agreements while in ICA [12] there is a wide spread of differences between the estimated density values and the ground truth (y-axis). This illustrates that by filtering the original OD image, correlation between the original sources can be reduced and thus sources are more independent and hence more separable.

**Fig 8:**
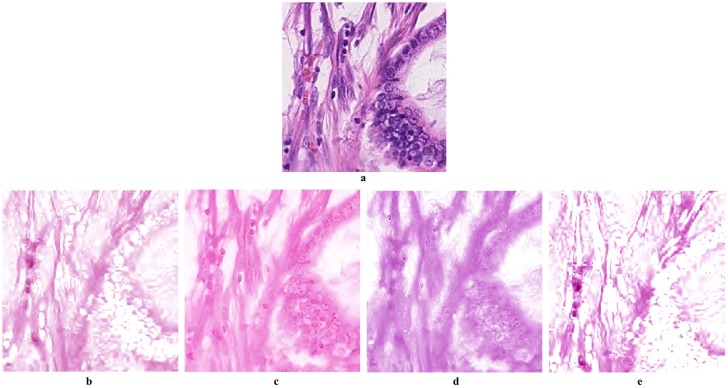
Estimation of Eosin channel for a sample image. Images a,b,c,d and e corresponds to the original image, Ruifrok and Johnston [10], Macenko *et al.* [11], BCD [13], respectively. We can notice in Ruifrok and Johnston method [10] that the pre-estimated mixing parameters is actually not reflecting the Eosin stain colour distribution in the original image. In Macenko *et al.* [11] method, the colour estimation is affected by the correlation between the two stain colours. In BCD method [13], the fine variation within the H stain is merged with the E due to the projection on the chromaticity plane. In the proposed method however, the variation of the stain colour distribution in the original image is perfectly reflected and H channel is smoothly separated.

It is worth noting here that density map and stain matrix estimation are interrelated to each other. In fact, inaccurate estimation of the stain matrix will result in the wrong stain colour and in that case the estimated density map is actually meaningless. In other words, without having the accurate stain matrix estimated, the density map does not actually represent the stain under observation. Therefore, we need to look to both factors when we are comparing stain deconvolution algorithms. An example of this is shown in Fig 4. One can notice that the estimated stain channels by [10–12] are far from the actual stain colours in the original image. The reason behind that is for [10], the stain matrix is pre-calculated and fixed for all images. In case of [13], projection on the Maxwellian chromaticity plane removes the small variations within one stain colour which results in a very rough separation between stain channels, see Fig 8 for a closer view of the deconvolution results.

#### Assessment of Tumour Nuclei Detection using the Estimated H channel

In this section we demonstrate that an accurate deconvolution for histology images could improve the performance in the further processing, such as nuclei detection. In this experiment, we evaluate the performance of the proposed stain deconvolution method using a nuclei detection algorithm proposed in [2, 28]. The algorithm uses a Spatially Constrained Convolutional Neural Network (SC-CNN) to detect the centroids of nuclei in colon histopathology images. The method uses the H and L*a*b* channels to find nuclei features. We use a sub-set of 20 images of the dataset and ground truth used in [2] to evaluate our method against the other published methods. For each of the evaluated algorithms, H channel is generated for all the images. Then, SC-CNN is re-trained using only the H channel for each algorithm separately. Results in Table 3 show that the proposed methods significantly improves the achieved F1 score compared to other method. Ruifrok and Johnston method [10] is not included in this experiments as the stain matrix is constant in all images and hence it is not reflecting the stability in stain colour estimation. Therefore, the factor that we are measuring here, i.e. stain colour consistency after the deconvolution, is not applicable to that method.

**Table 3:** Results of nuclei detection algorithm in [2, 28] trained and tested for different stain deconvolution algorithms. Values show the mean and standard deviation for each of the precision, recall, and F1 score measures. Note that the evaluated algorithms are dynamically estimating stain colour based on current information. Thus, consistency of the algorithm could improve the detection accuracy. However, we did not include stain normalization in this experiments to avoid affecting the deconvolution results.

	Precision	Recall	F1 score
**Proposed**	**0.809 ± 0.1972**	0.419±**0.2404**	**0.520**±**0.223**
**BCD** [13]	0.469±0.384	0.399±0.419	0.352±0.363
**Macenko** ***et al.*** [11]	0.407±0.281	**0.553** ±0.335	0.374±.0270
**ICA** [12]	0.370±0.351	0.416±0.262	0.288±0.240

## Conclusions

In this paper, we presented a novel method for stain deconvolution of histology images using multi-resolution wavelet representation of the image to estimate stain mixing matrix. We propose filtering the input image to allow the independent observations to pass through. We then use independent observations from the colour channels to estimate stain matrix which is not affected by the correlated signals. The estimated stain matrix is then applied to the raw image to find the individual stain colour distribution. We have shown through extensive experiments that the proposed algorithm outperforms the recent stain deconvolution algorithms. Our future direction would be customising the number of selected sub-bands to the image under process. Since images have different histology structure and the distribution of the stain colours are variable from one image to another, we can utilise this to allow dynamic estimation of the number of selected sub-bands and thus improve both computational and time complexity of our algorithm.

## Supporting Information

S1 File Data and code. This ZIP file contains all data and the MatLab code files for the proposed algorithm. Folder Data contains two folders: Folder GroundTruth contains the data used to perform the experiment and folder RGB-images contains all images used to generate data for experiments.
(ZIP)
