# Supplementary material for: Stain Deconvolution Using Statistical Analysis of Multi-Resolution Stain Colour Representation
Source: PLoS One. 2017 Jan 11;12(1):e0169875. doi: 10.1371/journal.pone.0169875 (PMC5226799; doi:10.1371/journal.pone.0169875)
Supplement: S1 File — This ZIP file contains all data and the MatLab code files for the proposed algorithm. Folder Data contains two folders: Folder GroundTruth contains the data used to perform the experiment and folder RGB-images contains all images used to generate data for experiments. (ZIP) [file pone.0169875.s001.zip › Stain-Deconvolution-using-StatisticalAnalysis_of-MultiresolutionStainColourRepresentatioN/README.rtf]

------------------------------------------------------------Stain Deconvolution using statistical analysis of multi-resolution stain color presentation Najah Alsubaie1,2 , Nicholas Trahearn1 , Shan E Ahmed Raza1 , David Snead3 , Nasir M.Rajpoot11  Department of Computer Science, University of Warwick, Coventry, UK2  Department of Computer Science, Princess Nourah University, Riyadh, KSA3  Department of Histopathology, University Hospitals Coventry and Warwickshire, Coventry, UK-------------------------------------------------------------4. Folder Contents-------------------------------------------------------------    The folder contains the following components:        (1) A MATLAB implementation of the Stain Deconvolution using statistical analysis of multi-resolution stain color presentation                Alsubaie N., Trahearn N., Raza S., Snead D. and Rajpoot N. , Stain Deconvolution using statistical analysis of multi-resolution stain color presentation,PloS ONE, 2016.         (2) MATLAB mat files for the generated ground truth stored in folder GroundTruth such that:             a. SV is the ground truth stain vector            b. number_H or number_E is the ground truth density maps.             c. Folder number is  GroundTruth matches the image number in RGB_images folder.-------------------------------------------------------------5. Notes-------------------------------------------------------------    The toolbox and its components are provided for research use only.    Please cite the paper if you use this code or any of the supplementary materials. -------------------------------------------------------------6. Contact Information-------------------------------------------------------------    Please send all comments and feedback to Najah Alsubaie at: n.m.f.alsuabie@warwick.ac.uk
