# Supplementary figures and images for: Stain Deconvolution Using Statistical Analysis of Multi-Resolution Stain Colour Representation
Source: PLoS One. 2017 Jan 11;12(1):e0169875. doi: 10.1371/journal.pone.0169875 (PMC5226799; doi:10.1371/journal.pone.0169875)

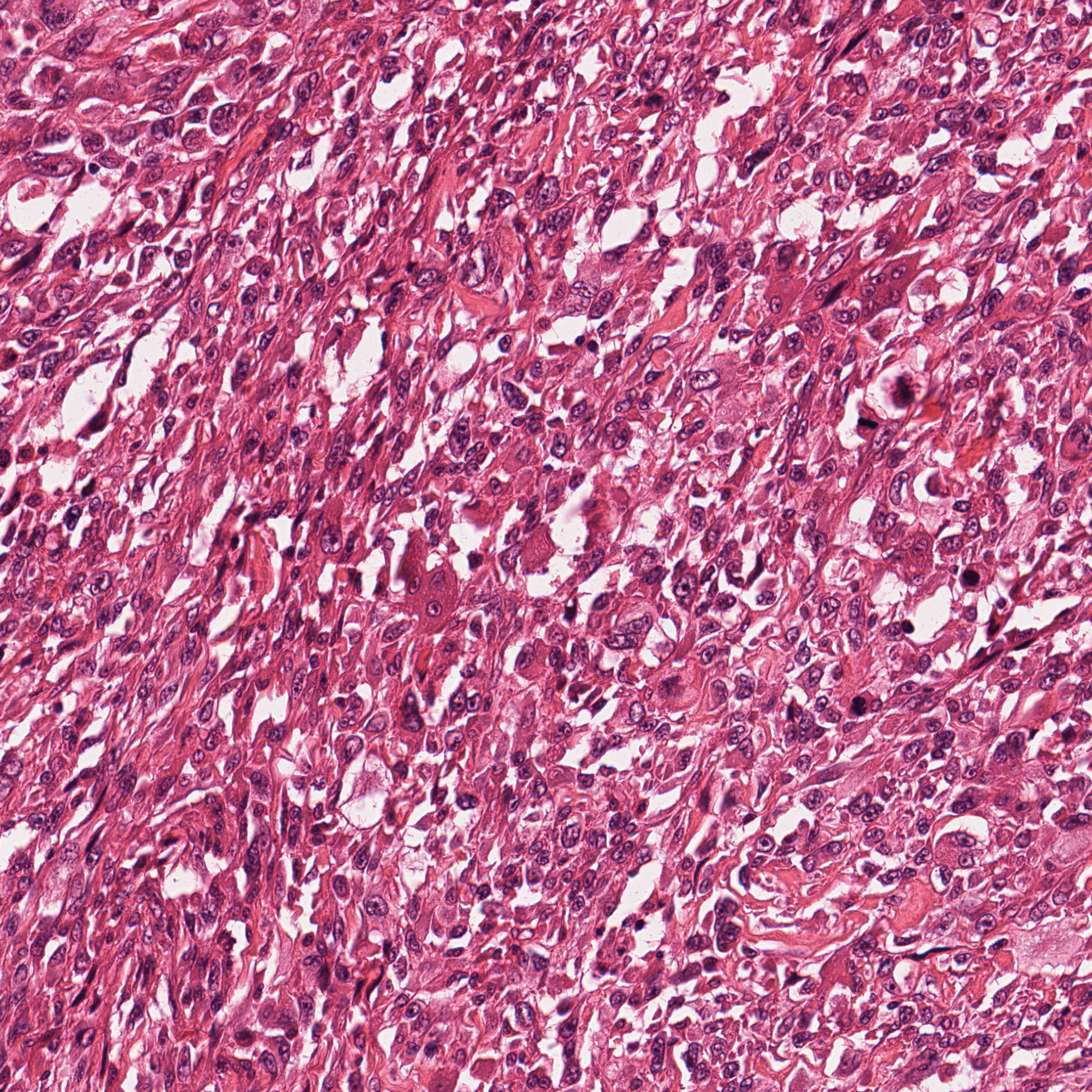

Supplement: S1 File — This ZIP file contains all data and the MatLab code files for the proposed algorithm. Folder Data contains two folders: Folder GroundTruth contains the data used to perform the experiment and folder RGB-images contains all images used to generate data for experiments. (ZIP) [file pone.0169875.s001.zip › Stain-Deconvolution-using-StatisticalAnalysis_of-MultiresolutionStainColourRepresentatioN/Data/RGB_images/Breast/1/01/01.png]

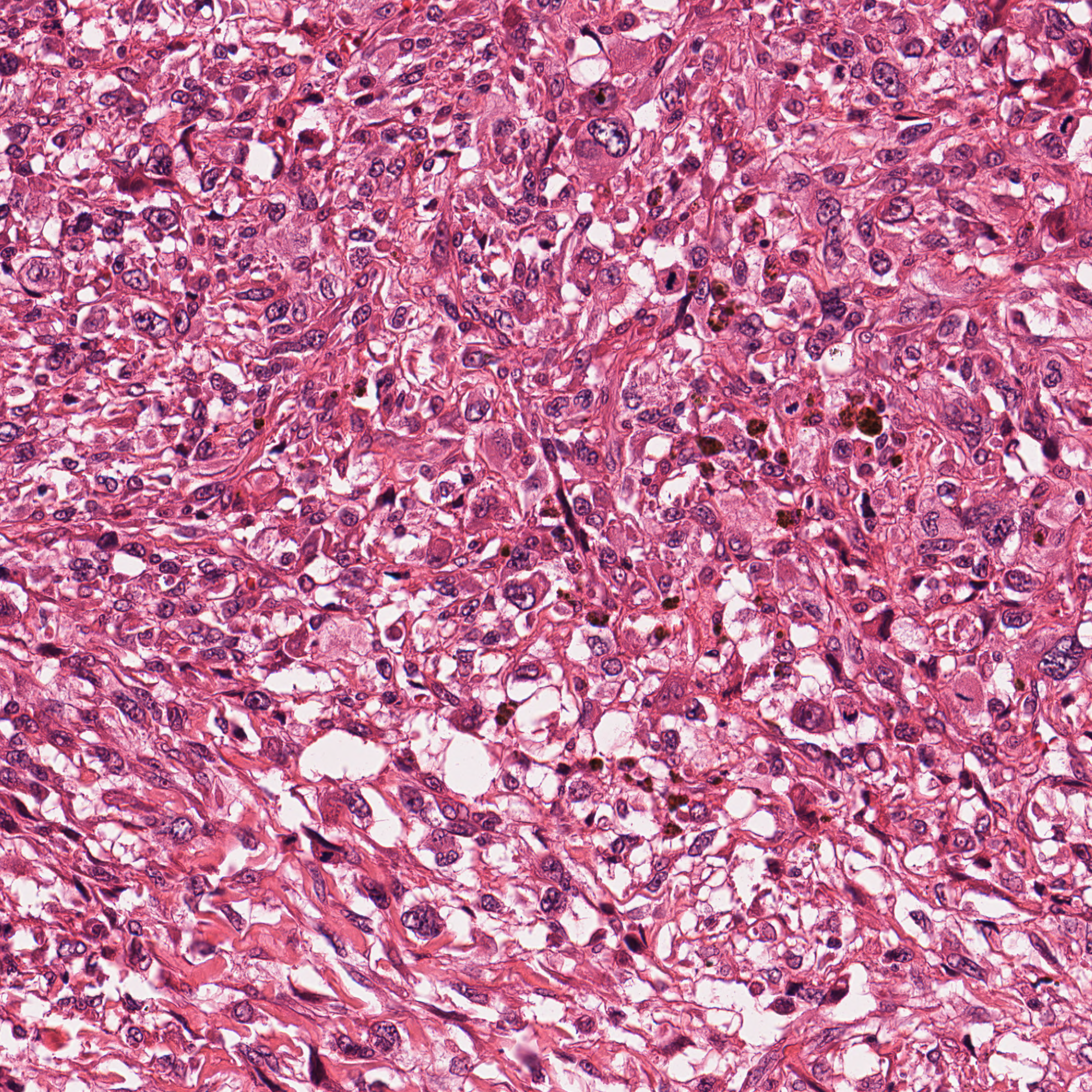

Supplement: S1 File — This ZIP file contains all data and the MatLab code files for the proposed algorithm. Folder Data contains two folders: Folder GroundTruth contains the data used to perform the experiment and folder RGB-images contains all images used to generate data for experiments. (ZIP) [file pone.0169875.s001.zip › Stain-Deconvolution-using-StatisticalAnalysis_of-MultiresolutionStainColourRepresentatioN/Data/RGB_images/Breast/1/02/02.png]

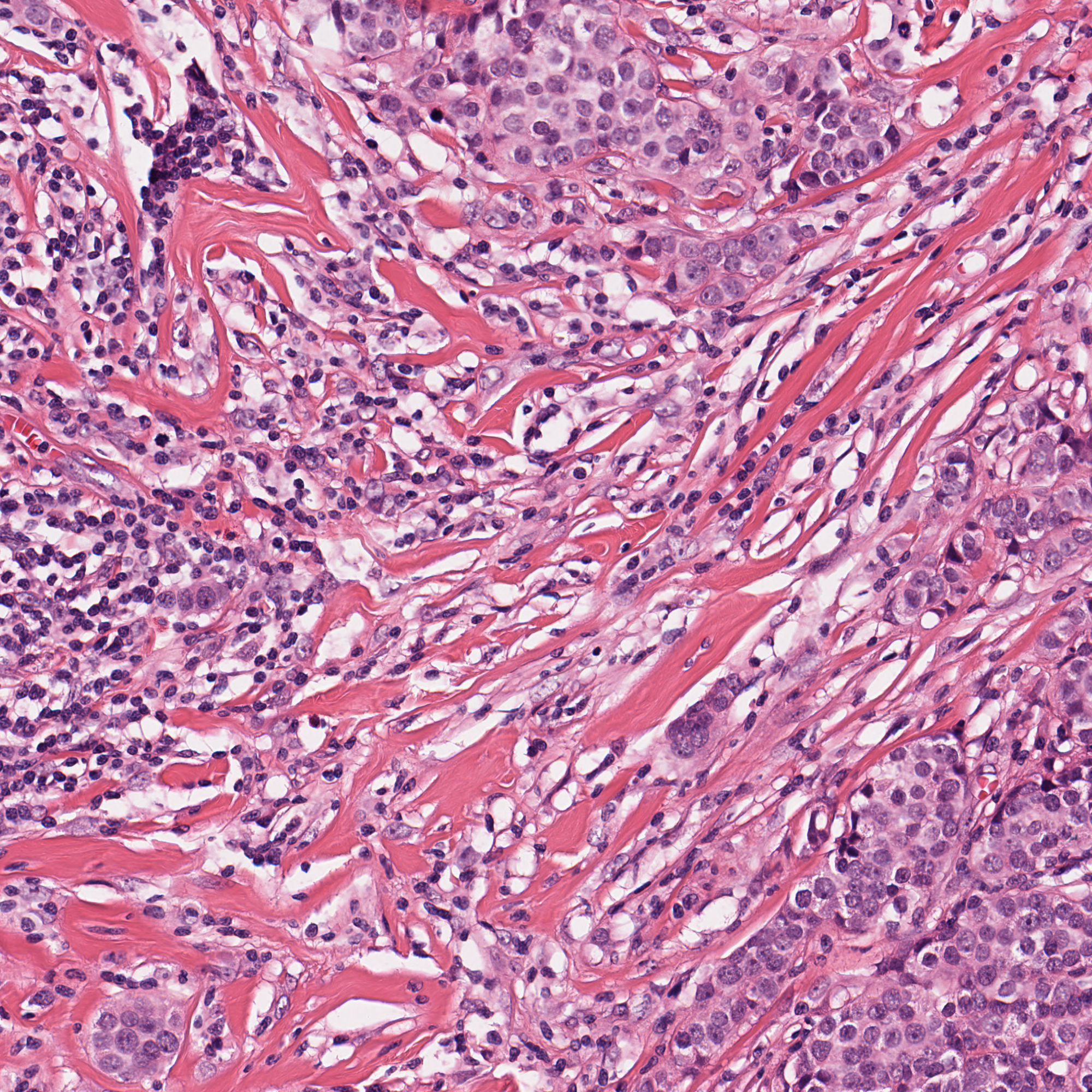

Supplement: S1 File — This ZIP file contains all data and the MatLab code files for the proposed algorithm. Folder Data contains two folders: Folder GroundTruth contains the data used to perform the experiment and folder RGB-images contains all images used to generate data for experiments. (ZIP) [file pone.0169875.s001.zip › Stain-Deconvolution-using-StatisticalAnalysis_of-MultiresolutionStainColourRepresentatioN/Data/RGB_images/Breast/2/03/03.png]

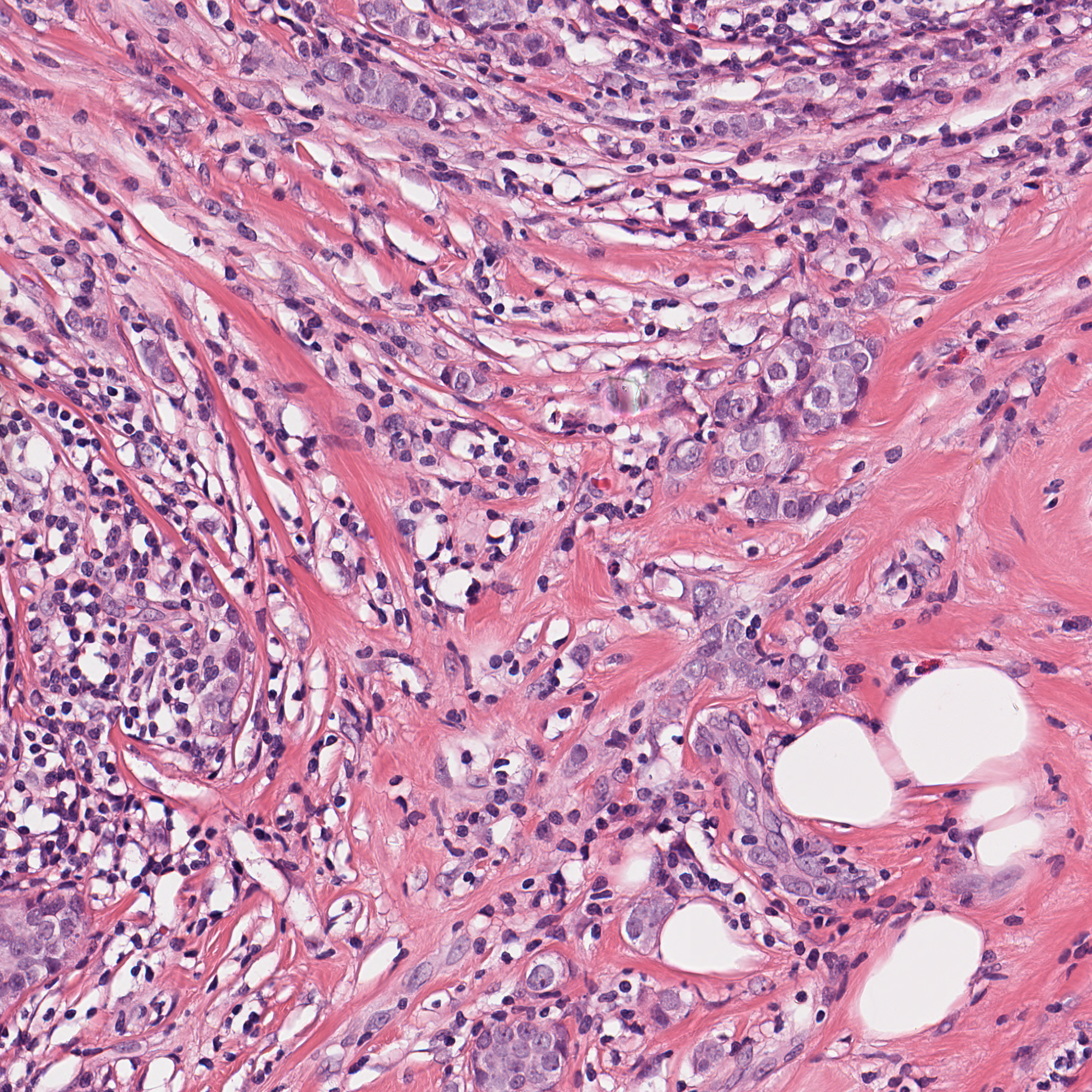

Supplement: S1 File — This ZIP file contains all data and the MatLab code files for the proposed algorithm. Folder Data contains two folders: Folder GroundTruth contains the data used to perform the experiment and folder RGB-images contains all images used to generate data for experiments. (ZIP) [file pone.0169875.s001.zip › Stain-Deconvolution-using-StatisticalAnalysis_of-MultiresolutionStainColourRepresentatioN/Data/RGB_images/Breast/2/08/08.png]

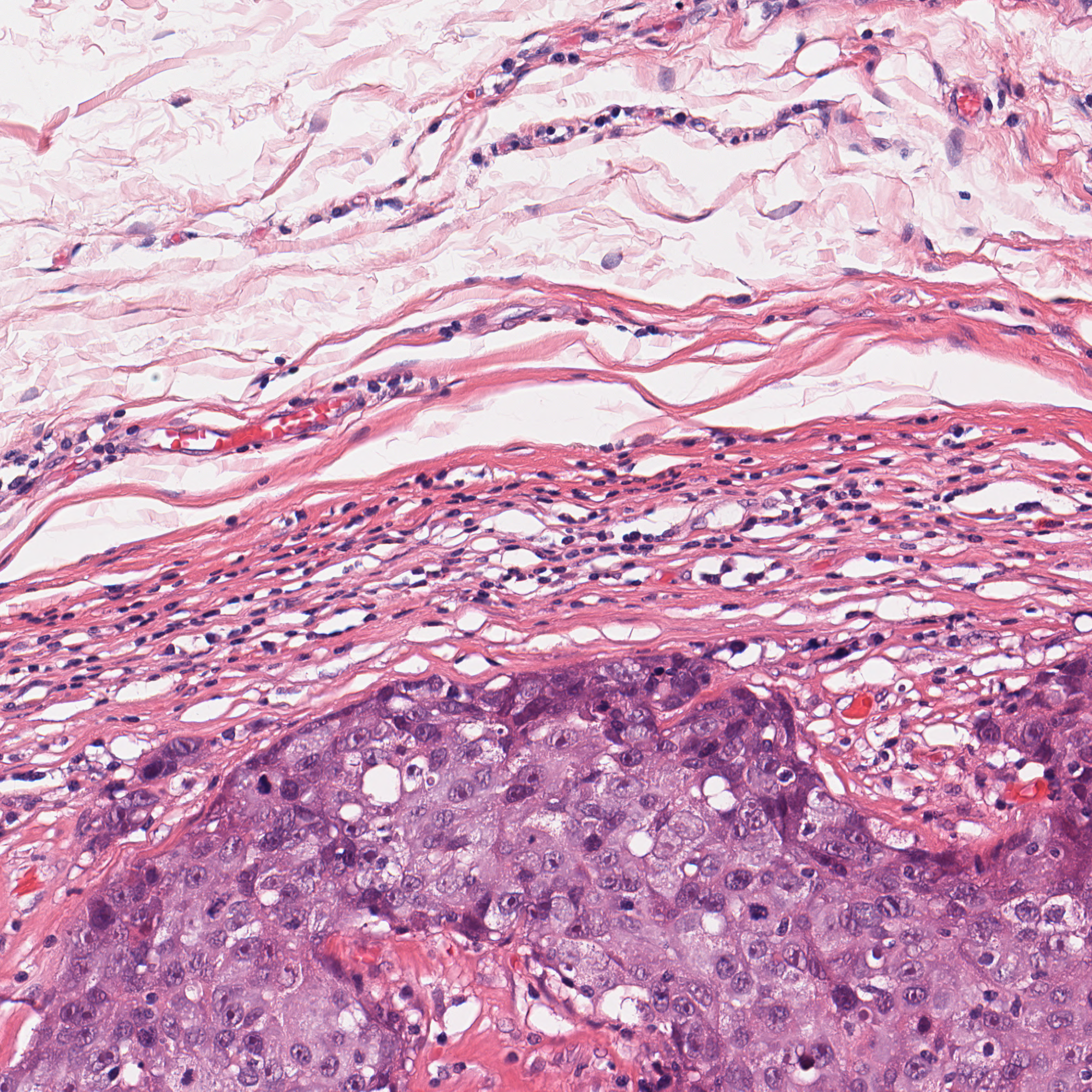

Supplement: S1 File — This ZIP file contains all data and the MatLab code files for the proposed algorithm. Folder Data contains two folders: Folder GroundTruth contains the data used to perform the experiment and folder RGB-images contains all images used to generate data for experiments. (ZIP) [file pone.0169875.s001.zip › Stain-Deconvolution-using-StatisticalAnalysis_of-MultiresolutionStainColourRepresentatioN/Data/RGB_images/Breast/3/01/01.png]

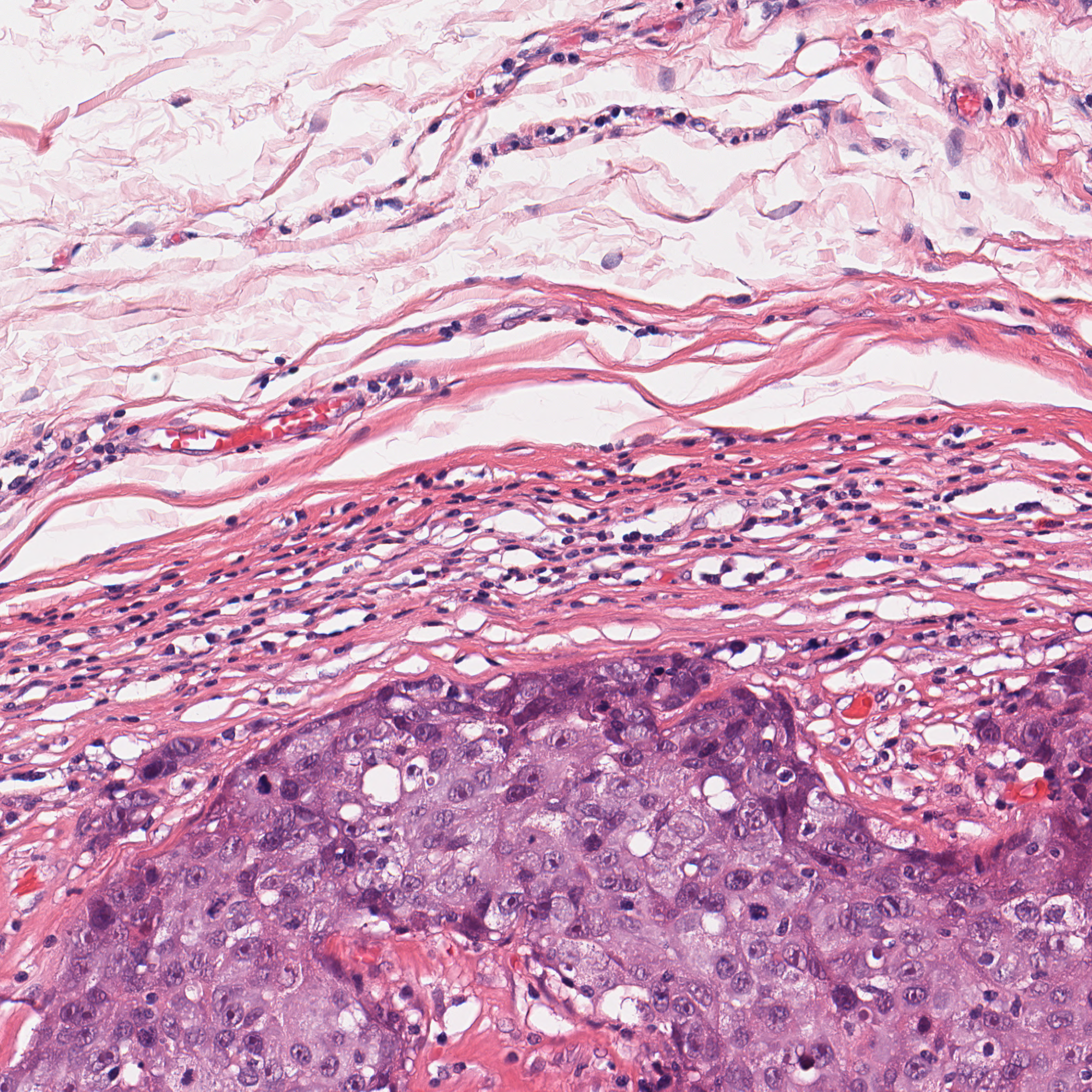

Supplement: S1 File — This ZIP file contains all data and the MatLab code files for the proposed algorithm. Folder Data contains two folders: Folder GroundTruth contains the data used to perform the experiment and folder RGB-images contains all images used to generate data for experiments. (ZIP) [file pone.0169875.s001.zip › Stain-Deconvolution-using-StatisticalAnalysis_of-MultiresolutionStainColourRepresentatioN/Data/RGB_images/Breast/3/01/01.tif]

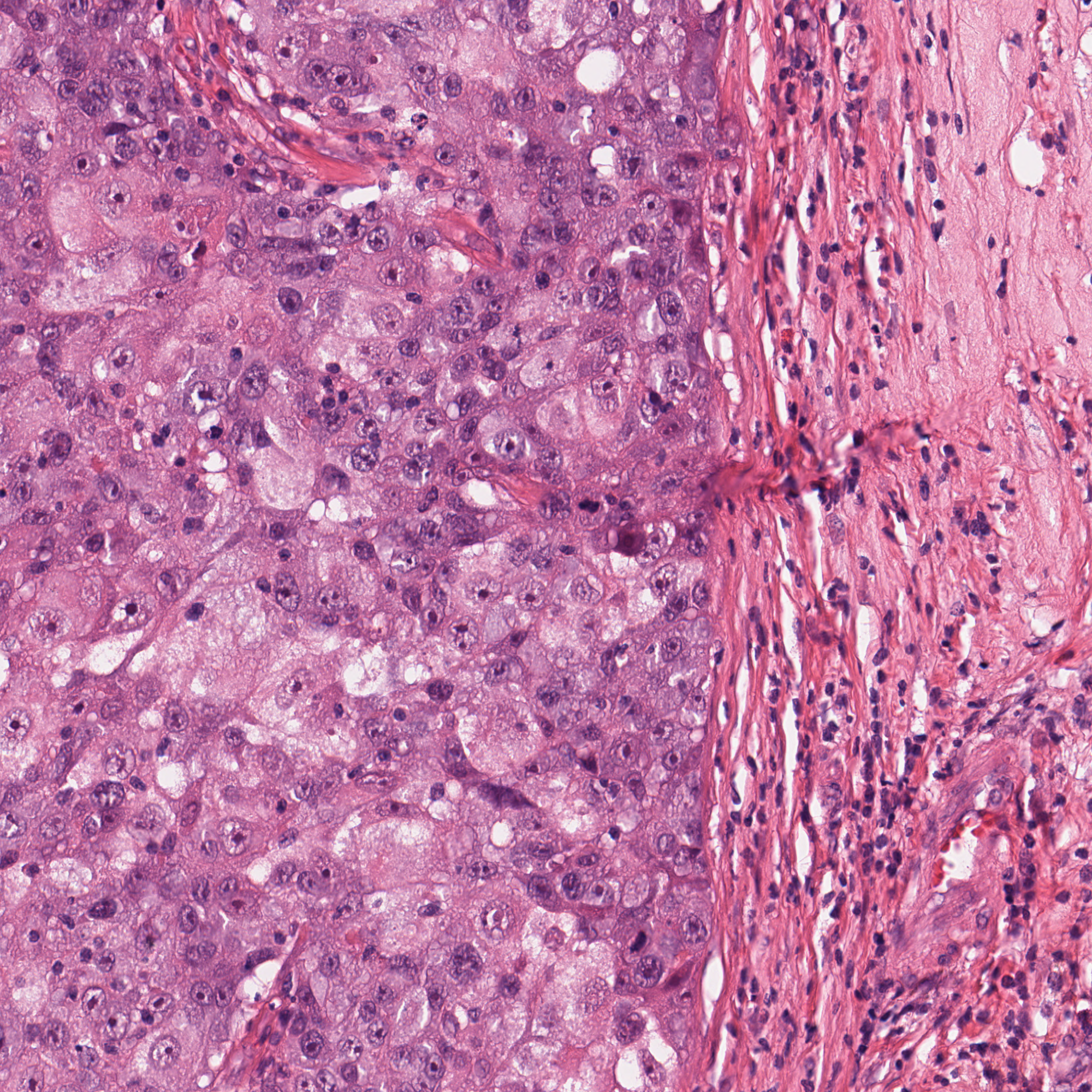

Supplement: S1 File — This ZIP file contains all data and the MatLab code files for the proposed algorithm. Folder Data contains two folders: Folder GroundTruth contains the data used to perform the experiment and folder RGB-images contains all images used to generate data for experiments. (ZIP) [file pone.0169875.s001.zip › Stain-Deconvolution-using-StatisticalAnalysis_of-MultiresolutionStainColourRepresentatioN/Data/RGB_images/Breast/3/06/06.png]

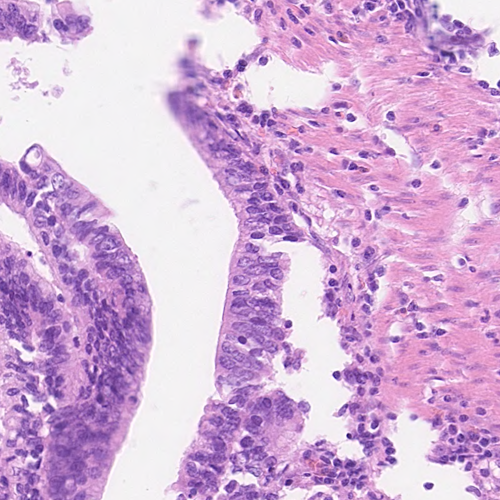

Supplement: S1 File — This ZIP file contains all data and the MatLab code files for the proposed algorithm. Folder Data contains two folders: Folder GroundTruth contains the data used to perform the experiment and folder RGB-images contains all images used to generate data for experiments. (ZIP) [file pone.0169875.s001.zip › Stain-Deconvolution-using-StatisticalAnalysis_of-MultiresolutionStainColourRepresentatioN/Data/RGB_images/colon/3/01/01.bmp]

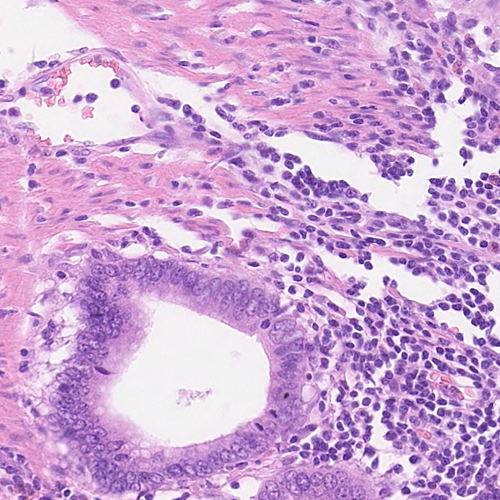

Supplement: S1 File — This ZIP file contains all data and the MatLab code files for the proposed algorithm. Folder Data contains two folders: Folder GroundTruth contains the data used to perform the experiment and folder RGB-images contains all images used to generate data for experiments. (ZIP) [file pone.0169875.s001.zip › Stain-Deconvolution-using-StatisticalAnalysis_of-MultiresolutionStainColourRepresentatioN/Data/RGB_images/colon/3/02/02.bmp]

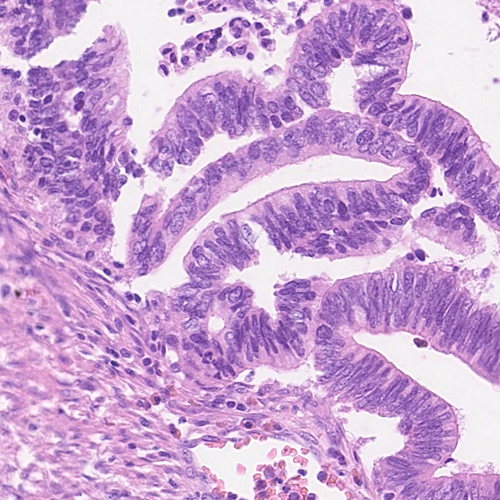

Supplement: S1 File — This ZIP file contains all data and the MatLab code files for the proposed algorithm. Folder Data contains two folders: Folder GroundTruth contains the data used to perform the experiment and folder RGB-images contains all images used to generate data for experiments. (ZIP) [file pone.0169875.s001.zip › Stain-Deconvolution-using-StatisticalAnalysis_of-MultiresolutionStainColourRepresentatioN/Data/RGB_images/colon/4/01/01.bmp]

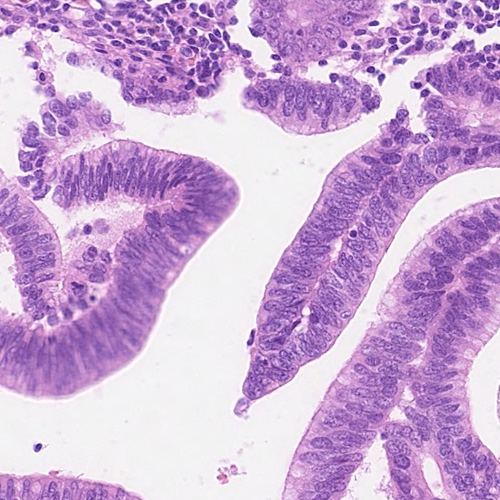

Supplement: S1 File — This ZIP file contains all data and the MatLab code files for the proposed algorithm. Folder Data contains two folders: Folder GroundTruth contains the data used to perform the experiment and folder RGB-images contains all images used to generate data for experiments. (ZIP) [file pone.0169875.s001.zip › Stain-Deconvolution-using-StatisticalAnalysis_of-MultiresolutionStainColourRepresentatioN/Data/RGB_images/colon/4/02/02.bmp]

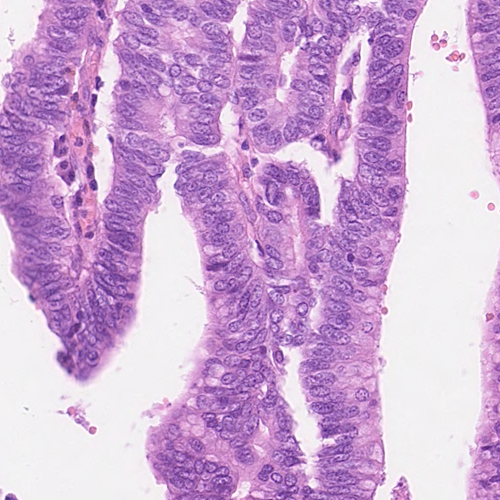

Supplement: S1 File — This ZIP file contains all data and the MatLab code files for the proposed algorithm. Folder Data contains two folders: Folder GroundTruth contains the data used to perform the experiment and folder RGB-images contains all images used to generate data for experiments. (ZIP) [file pone.0169875.s001.zip › Stain-Deconvolution-using-StatisticalAnalysis_of-MultiresolutionStainColourRepresentatioN/Data/RGB_images/colon/5/01/01.bmp]

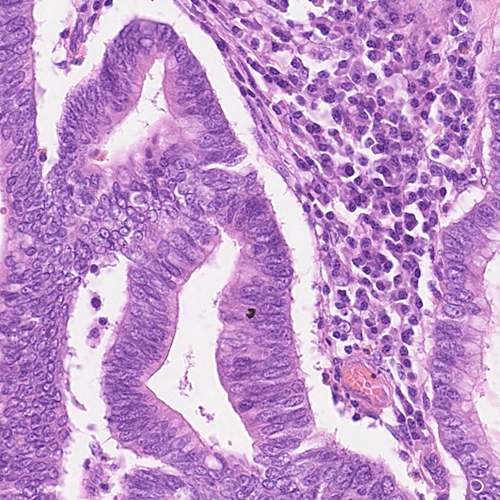

Supplement: S1 File — This ZIP file contains all data and the MatLab code files for the proposed algorithm. Folder Data contains two folders: Folder GroundTruth contains the data used to perform the experiment and folder RGB-images contains all images used to generate data for experiments. (ZIP) [file pone.0169875.s001.zip › Stain-Deconvolution-using-StatisticalAnalysis_of-MultiresolutionStainColourRepresentatioN/Data/RGB_images/colon/5/02/02.bmp]

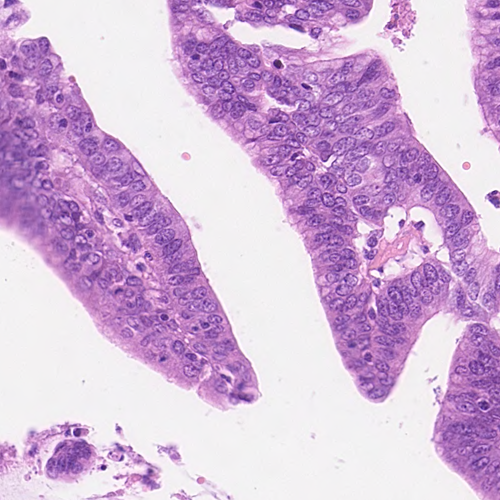

Supplement: S1 File — This ZIP file contains all data and the MatLab code files for the proposed algorithm. Folder Data contains two folders: Folder GroundTruth contains the data used to perform the experiment and folder RGB-images contains all images used to generate data for experiments. (ZIP) [file pone.0169875.s001.zip › Stain-Deconvolution-using-StatisticalAnalysis_of-MultiresolutionStainColourRepresentatioN/Data/RGB_images/colon/6/01/01.bmp]

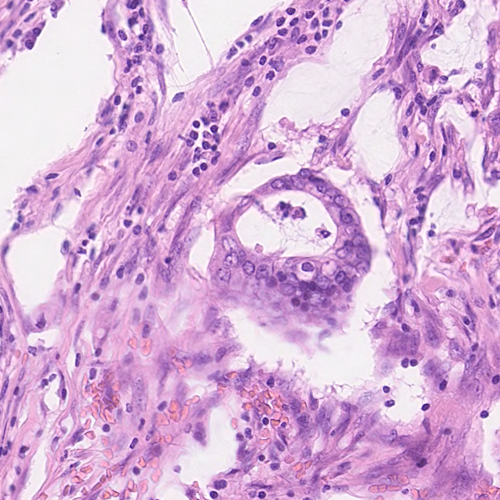

Supplement: S1 File — This ZIP file contains all data and the MatLab code files for the proposed algorithm. Folder Data contains two folders: Folder GroundTruth contains the data used to perform the experiment and folder RGB-images contains all images used to generate data for experiments. (ZIP) [file pone.0169875.s001.zip › Stain-Deconvolution-using-StatisticalAnalysis_of-MultiresolutionStainColourRepresentatioN/Data/RGB_images/colon/7/01/01.bmp]

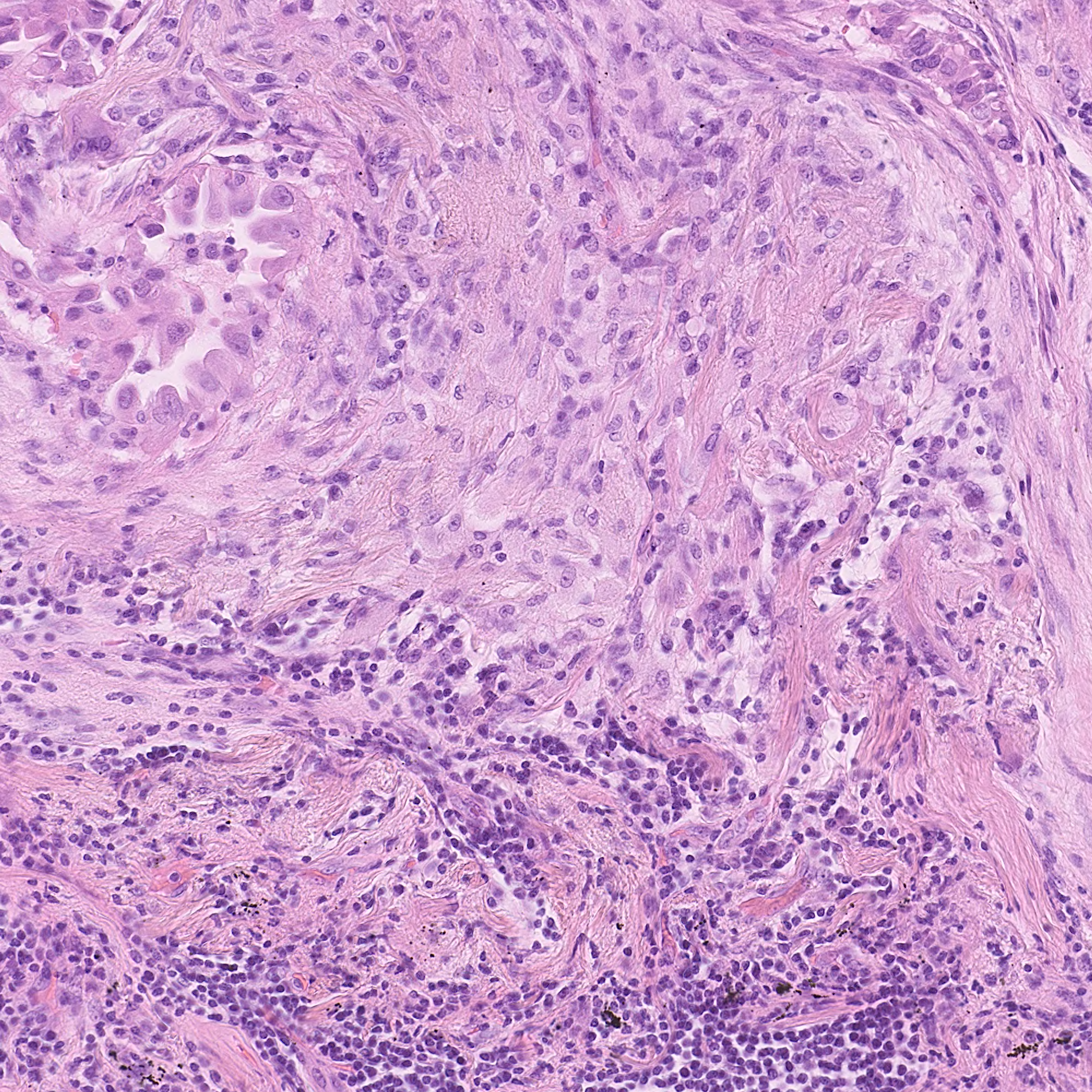

Supplement: S1 File — This ZIP file contains all data and the MatLab code files for the proposed algorithm. Folder Data contains two folders: Folder GroundTruth contains the data used to perform the experiment and folder RGB-images contains all images used to generate data for experiments. (ZIP) [file pone.0169875.s001.zip › Stain-Deconvolution-using-StatisticalAnalysis_of-MultiresolutionStainColourRepresentatioN/Data/RGB_images/Lung/1/01/01.png]

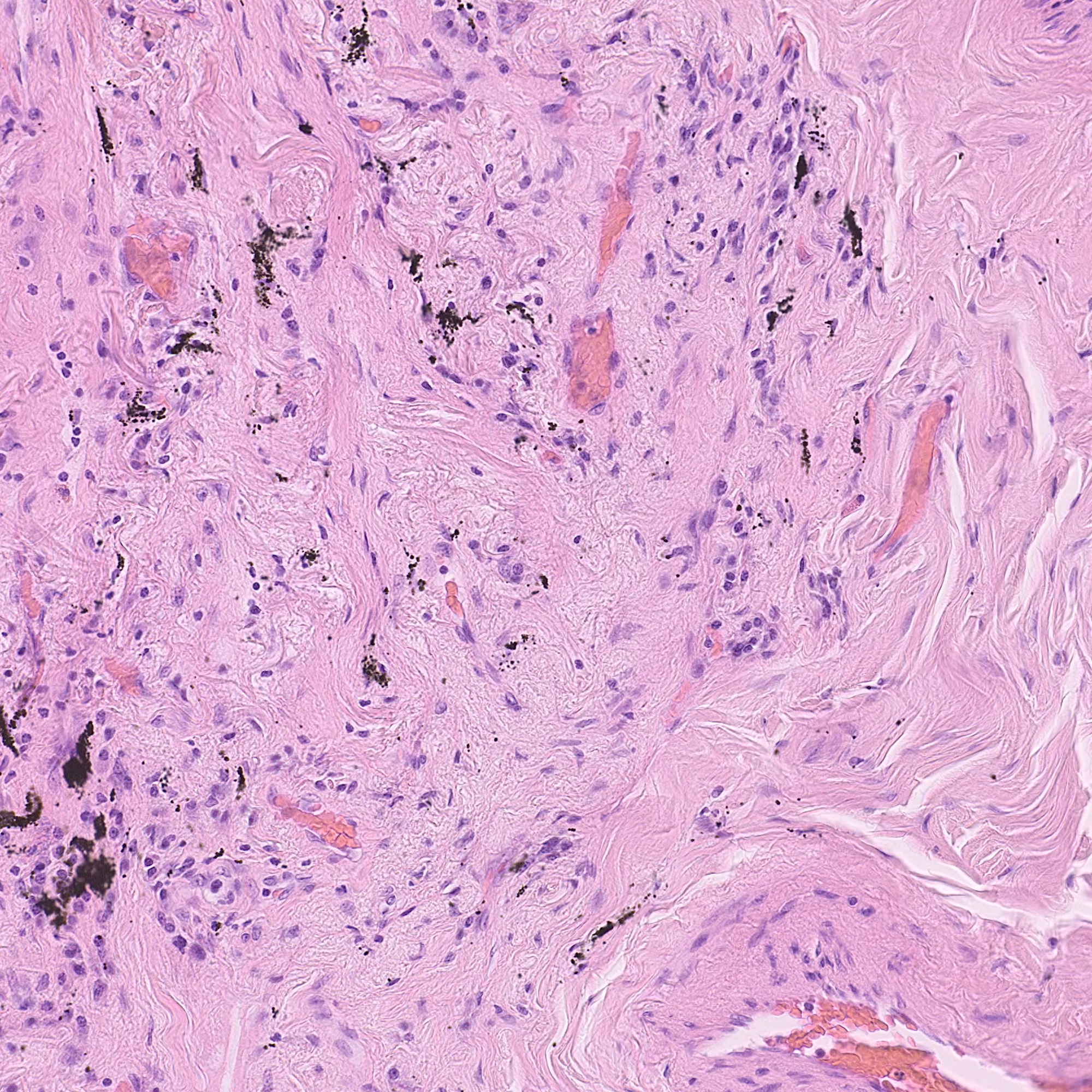

Supplement: S1 File — This ZIP file contains all data and the MatLab code files for the proposed algorithm. Folder Data contains two folders: Folder GroundTruth contains the data used to perform the experiment and folder RGB-images contains all images used to generate data for experiments. (ZIP) [file pone.0169875.s001.zip › Stain-Deconvolution-using-StatisticalAnalysis_of-MultiresolutionStainColourRepresentatioN/Data/RGB_images/Lung/1/02/02.png]

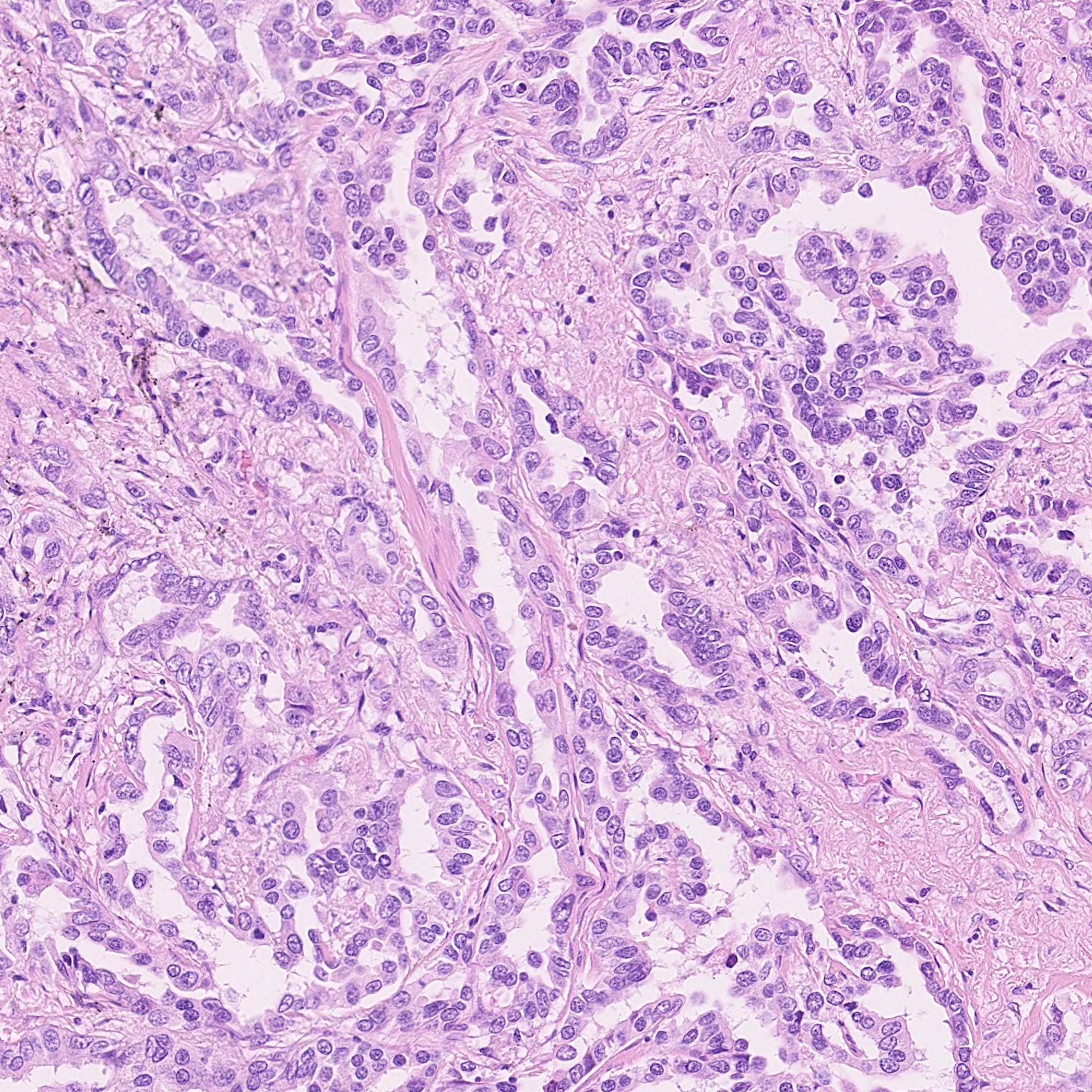

Supplement: S1 File — This ZIP file contains all data and the MatLab code files for the proposed algorithm. Folder Data contains two folders: Folder GroundTruth contains the data used to perform the experiment and folder RGB-images contains all images used to generate data for experiments. (ZIP) [file pone.0169875.s001.zip › Stain-Deconvolution-using-StatisticalAnalysis_of-MultiresolutionStainColourRepresentatioN/Data/RGB_images/Lung/2/01/01.png]

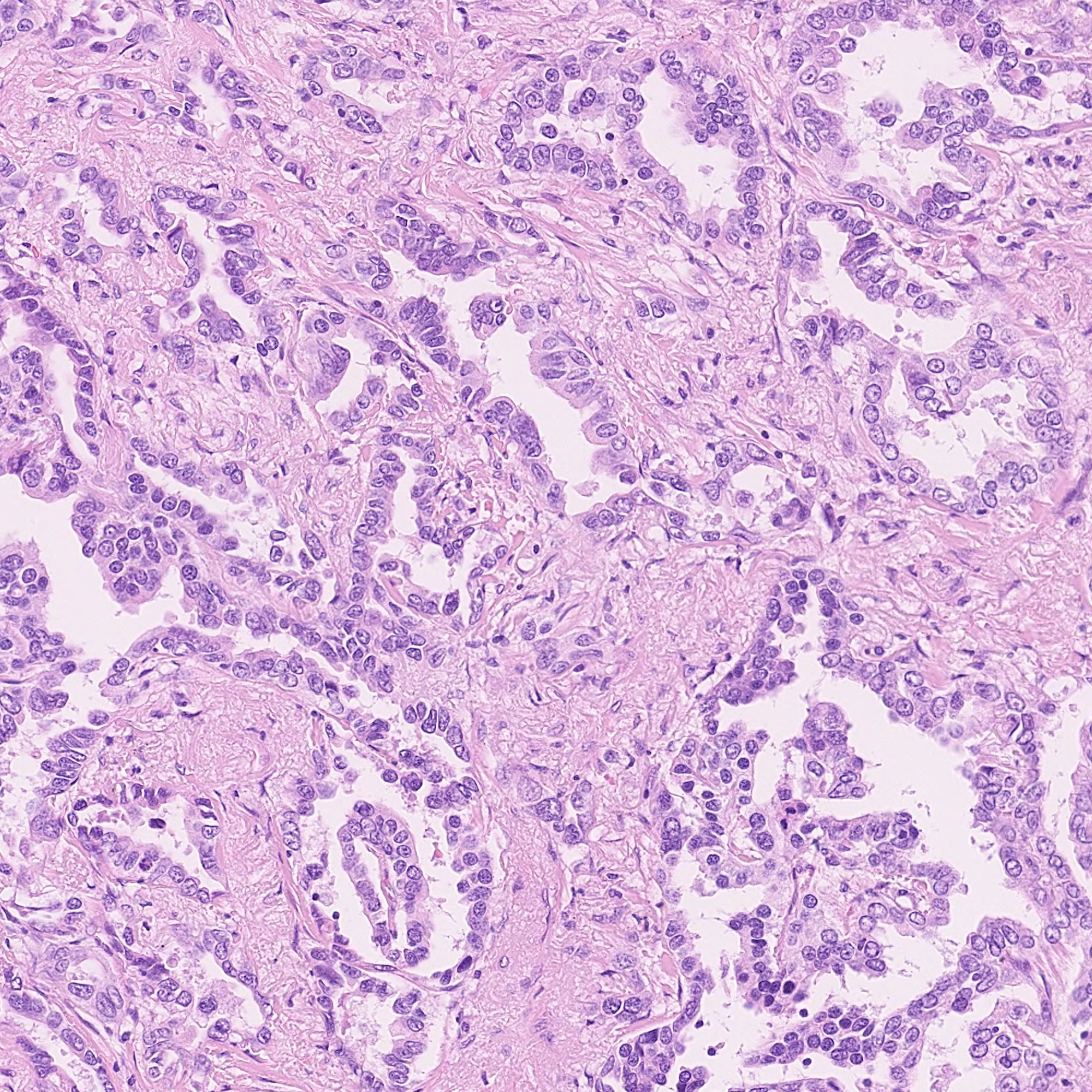

Supplement: S1 File — This ZIP file contains all data and the MatLab code files for the proposed algorithm. Folder Data contains two folders: Folder GroundTruth contains the data used to perform the experiment and folder RGB-images contains all images used to generate data for experiments. (ZIP) [file pone.0169875.s001.zip › Stain-Deconvolution-using-StatisticalAnalysis_of-MultiresolutionStainColourRepresentatioN/Data/RGB_images/Lung/2/02/02.png]
